# Hsp40/JDP Requirements for the Propagation of Synthetic Yeast Prions

**DOI:** 10.3390/v14102160

**Published:** 2022-09-30

**Authors:** Sarah C. Miller, Andrea K. Wegrzynowicz, Sierra J. Cole, Rachel E. Hayward, Samantha J. Ganser, Justin K. Hines

**Affiliations:** Department of Chemistry, Lafayette College, Easton, PA 18042, USA

**Keywords:** J-domain protein, Hsp40, Sis1, yeast prion, molecular chaperone, amyloid, polygulatamine

## Abstract

Yeast prions are protein-based transmissible elements, most of which are amyloids. The chaperone protein network in yeast is inexorably linked to the spreading of prions during cell division by fragmentation of amyloid prion aggregates. Specifically, the core “prion fragmentation machinery” includes the proteins Hsp104, Hsp70 and the Hsp40/J-domain protein (JDP) Sis1. Numerous novel amyloid-forming proteins have been created and examined in the yeast system and occasionally these amyloids are also capable of continuous Hsp104-dependent propagation in cell populations, forming synthetic prions. However, additional chaperone requirements, if any, have not been determined. Here, we report the first instances of a JDP-Hsp70 system requirement for the propagation of synthetic prions. We utilized constructs from a system of engineered prions with prion-forming domains (PrDs) consisting of a polyQ stretch interrupted by a single heterologous amino acid interspersed every fifth residue. These “polyQX” PrDs are fused to the MC domains of Sup35, creating chimeric proteins of which a subset forms synthetic prions in yeast. For four of these prions, we show that *SIS1* repression causes prion loss in a manner consistent with Sis1′s known role in prion fragmentation. PolyQX prions were sensitive to Sis1 expression levels to differing degrees, congruent with the variability observed among native prions. Our results expand the scope known Sis1 functionality, demonstrating that Sis1 acts on amyloids broadly, rather than through specific protein–protein interactions with individual yeast prion-forming proteins.

## 1. Introduction

### 1.1. Native Prions and Chaperone Proteins

Many human and livestock diseases, including Bovine Spongiform Encephalopathy (Mad Cow Disease), Creutzfeld-Jakob, and sheep scrapie, are caused by the prion protein PrP in its amyloid form [1]. The prion phenomenon is not limited to mammals, and numerous proteins have been discovered in the baker’s yeast *Saccharomyces cerevisiae* that form self-templating, heritable aggregates [2,3,4,5,6,7]. The most studied yeast prions, [*PSI^+^*], [*PIN*^+^]/[*RNQ^+^*], [*URE3*], and [*SWI*^+^] are the amyloid forms of the proteins Sup35, Rnq1, Ure2, and Swi1, respectively [8], and have become useful tools not only for studying yeast prion characteristics and propagation, but also understanding chaperone protein networks in both yeast and higher organisms [9,10,11,12,13,14,15].

Chaperone proteins, which normally participate in protein folding and proteostasis, are required for propagation of yeast prions. To be maintained in the cell population, prion aggregates must be fragmented and the smaller pieces, called propagons, transmitted to daughter cells during mitosis. This fragmentation process is carried out by a set of at least three chaperone proteins: the Hsp100 Hsp104, the Hsp70 Ssa, and the Hsp40/JDP Sis1 [8]. Hsp40s were previously called J-proteins and are now preferably termed J-domain proteins or JDPs [16]. Hsp104 is required for the fragmentation of all amyloid-based yeast prions, most easily demonstrated by loss of the prion in the population (“curing”) after inhibition of Hsp104 by guanidine hydrochloride (GdnHCl) [17,18,19]. Sis1 is thought to first recruit Ssa, which then recruits Hsp104 [20], and fragmentation is accomplished by the action of Hsp104 [8,21].

Prions manifest as distinct structural conformers known as strains in mammalian systems and variants in yeast, first clearly noted in yeast by Derkatch et al. for the prion [*PSI*^+^] [4]. While the roles of Hsp104 and the Hsp70 Ssa appear to be universal in prion propagation, because different prions and even prion variants demonstrate varied requirements for one or more JDPs, these proteins are thought to be the prion-specific recognition factor in the propagation machinery (recently reviewed in Killian and Hines 2018) [22]. There are 13 JDPs in the yeast cytosol [23], and several have been implicated in prion propagation. In particular, Sis1 is required for all four of the best-studied yeast prions, [*PSI^+^*], [*RNQ^+^*], [*URE3*], and [*SWI*^+^] [24,25,26], while the JDPs Swa2 and Ydj1 are additionally required for propagation of [*URE3*] and [*SWI*^+^], respectively [25,27]. Different prions and prion variants sometimes even require different specific domains of these JDPs, further supporting the idea of JDPs as conformer-specific recognition factors [28,29,30].

### 1.2. Amino Acid Composition of Prion-Forming Domains

Yeast prion amyloid fibrils are formed by a specific region of the protein, termed the prion-forming domain (PrD) [4,31,32,33,34]. The PrDs of several prions may be scrambled and still retain their amyloid-forming abilities, indicating that overall amino acid composition, rather than primary sequence, is the main determinant of prion-forming propensity of a given protein [35,36]. In general, charged residues and proline are not found often in PrDs. Hydrophobic amino acids technically demonstrate a high prion-forming propensity, but too many hydrophobic residues may result in a highly stable native fold rather than amyloid formation and can enhance protein degradation [37,38]. Yeast PrDs therefore lack many bulky hydrophobic residues, and instead are richer in polar uncharged amino acids [39]. Particularly, glutamine (Q) and asparagine (N) are overrepresented in yeast PrDs, presumably to strike a balance between sufficient prion-forming propensity and the tendency to promote protein degradation [38,39].

Specific amino acids within a PrD can promote fragmentation. Tyrosine was first identified [40], followed by phenylalanine and tryptophan, all of which support efficient fragmentation and prion maintenance [41,42]. While nonaromatic hydrophobic residues theoretically increase the likelihood of prion formation, they score poorly in prion maintenance propensity algorithms. This suggests they do not support propagation, further explaining why they are not commonly found in yeast PrDs [42]. Indeed, the yeast prion [*PSI^+^*], one of the most studied and most efficiently fragmented prions, embodies most of these characteristics, as its monomeric form Sup35 has a PrD rich in Q and N, and with a higher frequency of tyrosine residues than other studied yeast prions [43]. Additionally, [*PSI^+^*] and the prion [*RNQ^+^*], when compared to others, exhibit higher Q/N ratios, have the fewest large nonaromatic hydrophobic residues, and have the greatest number of prion propagons [43]. This again suggests that the more efficiently fragmented yeast prions are those that have slightly less amyloidogenic PrDs, perhaps due to relative weakness of the amyloid core or due to increased recognition by chaperone proteins [22,43].

### 1.3. Synthetic Polyglutamine Models of Prion Propagation and Human Diseases

Many proteins in yeast and mammals have extended glutamine tracts, several of which are associated with diseases. The best known is the huntingtin protein (Htt), responsible for the neurodegenerative disorder Huntington’s disease [44]. Long polyQ stretches are able to aggregate, with the likelihood of aggregation increasing with a larger number of Q residues [40,45]. In fact, the length of the CAG repeat section an individual’s *HTT* gene can be used as a predictor of disease onset and phenotype [44].

Based on the high frequency of glutamine within the PrD regions of yeast prions, there has been interest in crafting synthetic constructs rich in glutamine to explore the variables in amyloid formation and prion propagation. For example, in 1998 DePace et al. replaced a small portion of the N-terminal region of Sup35 with a polyQ stretch and the resulting PrD retained its ability to form new aggregates and to be recruited into existing aggregates [46]. Osherovich et al. later designed novel synthetic prions through the fusion of expanded polyQ regions of proteins with portions of Sup35 [47], demonstrating the possibility for further experiments with synthetic Sup35 constructs. Htt constructs have also been interrogated in yeast. For example, Krobitsch and Lindquist expressed an N-terminal fragment of Htt in yeast cells with varied polyQ lengths, fused to a green fluorescent protein which enabled them to observe the relationship between polyQ length and aggregation [45]. They found that the extent of aggregation varied with polyQ length, with the shorter polyQ repeat of 25 (25Q) resulting in no aggregation and the longer stretches of 103Q resulted in a large cytoplasmic aggregate, with smaller aggregates forming from medium polyQ lengths [45].

**Figure 1:**
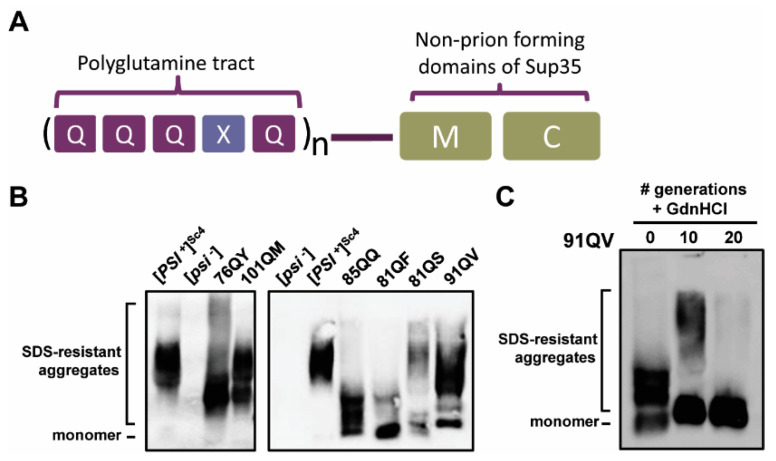
PolyQX-Sup35MC chimeras form stable Hsp104-dependent synthetic prions in yeast. (**A**) Gene structure diagrams of polyQX expression constructs. An N-terminal polyQ domain with a single residue interspersed every 5th residue is fused to the M and C domains of Sup35 as described in Alexandrov et al. [41]. In text and figures, constructs are denoted as in Alexandrov et al. by the overall length of the QQQXQ repeat region which sometimes ends with an additional terminal Q, followed by the letter Q and single letter code for the heterologous residue, e.g., 101QM. (**B**) PolyQX proteins form stable prions with detergent-resistant aggregates detectable by semi-denaturing detergent agarose gel electrophoresis (SDD-AGE) analysis. Lysates of *sis1*-Δ [*TETr-Sis1*] cells with various polyQX prions were resolved by SDD-AGE and visualized by immunoblotting using antibody specific for Sup35 which recognizes the identical C-terminal domain of each construct. Note that two separate blots are shown with wild-type control [*PSI*^+^] and [*psi*^−^] cells for each variant included for comparison on each. (**C**) The six polyQX prions used here are Hsp104 dependent as described for 76QF in Alexandrov et al. [41]. A representative gel for the curing of 91QV is shown. 76QY, 101QM, 85QQ, 81QF, and 81QS were also tested with similar results. Cells were cultured in rich liquid media with aeration for at least four generations in log growth before the addition of 4 mM GdnHCl (generation = 0). Cells were maintained in log growth at 30 °C for 20 generations and cell pellets collected at 0, 10, and 20 generations for analysis by SDD-AGE followed by immunoblotting with Sup35 antibody.

Considering the association of polyQ tracts with disease, as well as the propensity of Q to aggregate in both yeast prions and other proteins, there is great appeal to using polyglutamine in model systems to understand the fundamental biochemistry underlying both human disease and yeast prion biology. The former Ter-Avanesyan group at the Russian Academy of the Sciences used polyQ tracts fused to the C-terminal, non-prion forming regions of yeast Sup35 (Sup35MC) for studies of aggregate formation and propagation. Therefore, replacing the entire N-terminal PrD with polyQ tracts tests the ability of these polyQ tracts to serve as yeast “model PrDs”, and creates a tractable model with similar characteristics to those used to investigate [*PSI*^+^] formation and maintenance [40]. These polyQ tracts aggregate when the tract is longer than 70 residues and do not display any variants, likely due to the lack of sequence complexity. The group also demonstrated that if a single other amino acid is interspersed every fifth residue within the polyQ tract (Figure 1A), several constructs form aggregates that are SDS-resistant and dependent upon Hsp104 for propagation, meaning they are highly similar to yeast prions. Different aggregate sizes were noted, with smaller aggregates presumed to indicate that the aggregates were more efficiently fragmented and maintained [41].

The Ter-Avanesyan group’s set of polyQX-Sup35MC constructs, hereafter referred to as “polyQX”, the X referring to the heterogeneous amino acid in the particular construct, provides a model of amyloid formation with highly controlled amino acid composition. The impact of specific amino acids on prion formation and propagation is an important and ongoing area of study, but the mechanisms of these impacts are not at all clear. Alexandrov et al. already demonstrated the involvement of Hsp104 in the propagation of one polyQX constructs, suggesting the entire Hsp104/Hsp70/JDP system may be required [41], but it remains unknown whether JDPs and their commensurate Hsp70 partners may be acting on polyQX aggregates. Thus, we endeavored to test whether specific constructs from this system might also rely upon recognition by JDPs for propagation like native yeast prions.

## 2. Materials and Methods

### 2.1. Yeast Strains and Plasmids

A [*pin*^−^] version of haploid *Saccharomyces cerevisiae* strain of the 74D-694 background, EAC Y1226, a gift from the Craig lab which expresses Sis1-121 from the plasmid [*SIS1*-Sis1-121, *TRP1*], was transformed with plasmid expressing full-length Sis1 and the *URA3* gene ([*SIS1*-Sis1, *URA3*]). The *TRP1*-marked plasmid was shuffled out using counterselection on medium containing 5-fluoroanthranilic acid (5-FAA). The strain was then transformed separately with both the Sis1 plasmid p3617 ([*TETr*-Sis1, *TRP1*]), a *TRP1*-marked tetracycline-repressible Sis1 plasmid, and p1849, a *TRP1*-marked constitutive Sis1 plasmid. The *URA3*-marked plasmid was then shuffled out using counterselection on medium containing 5-fluoroorotic acid (5-FOA). These strains were then transformed with the *URA3*-marked polyQX plasmids (gifted by the Ter-Avanesyan lab; see Alexandrov et al. 2012) individually, and transformants selected on –Ura medium. Approximately 10 transformants were then repatched and allowed to grow at 30 °C for 3 days on –Ura medium. Each transformant patch was then streaked to single colonies, and individual colonies repatched again and then analyzed by SDD-AGE to confirm aggregation of the polyQX constructs. Both initially, and in the course of our investigation, we repeatedly observed cell populations with and without SDS-resistant aggregates, indicating that distinct “prion” and “non-prion” states exist.

For Swa2 experiments, two W303 haploid *Saccharomyces cerevisiae* strains EAC Y1928 (Craig lab) and PD24 (Lindquist lab) were crossed, and zygotes grown on –His medium to maintain plasmid expressing the C-terminal domain of Sup35 ([*SUP35*-Sup35C, *HIS3*,]) [6]. Diploids were selected and sporulated on –His potassium acetate medium. After 3–4 days, haploids were tested for hygromycin B resistance (to maintain *sup35::HygBR*), His prototrophy, and mating type. A haploid strain, with genotype *MAT alpha*, *ade1-14*, *ADE2, met2-Δ1*, *trp1-1*, *ura3-1*, *leu2-3,112*, *his3-11,15*, *can1-100*, *lys2-Δ2*, *GAL2+*, *sup35::HygBr,* [*SUP35*-Sup35C, *HIS3*,], [*pin*^−^], was renamed J102. J102 was transformed with the polyQX plasmids, and the transformants repatched on –Ura media to allow for loss of the *HIS3*-marked plasmid. PolyQX aggregation was confirmed by SDD-AGE. These [*pin*^−^] polyQX strains were crossed with EAC Y1972, a [*pin*^−^] *swa2*::*HIS3* strain. Diploids were sporulated and spores were selected for hygromycin resistance and uracil prototrophy and then binned into two groups: those that were prototrophic for histidine (swa2-Δ) and those that were auxotrophic for histidine (*SWA2*). SDD-AGE was then carried out for each polyQX strain to test for the presence of polyQX aggregates.

The plasmids bearing *TETrSIS1* or *sis1*Δ*G/F*, as well as other plasmids used for plasmid shuffling experiments were described previously [48,49,50], and all are based on the pRS plasmid series [51].

### 2.2. SDD-AGE, SDS-PAGE, and Immunoblot Analyses

Semi-denaturing detergent agarose gel electrophoresis (SDD-AGE) was used to resolve detergent-resistant aggregates. Cells were grown in 5 mL –Ura medium and 4–8 OD_600_ of cells were collected by centrifugation at 14,500 rpm in a tabletop centrifuge for 1 min. Supernatants were discarded and cells were resuspended in 200 µL of protein extraction buffer (25 mM Tris-HCl (pH 7.5), 100 mM NaCl, 10 mM MgCl_2_, 1 mM DTT, 1 mM EDTA, in 50 mL DI solution) with sterile glass beads. Cells were lysed by bead-beating with 5 cycles of 1 min of vortexing and 1 min at rest, at 4 °C, and then centrifuged at 14,500 rpm on a tabletop centrifuge for 5 min at 4 °C. Lysates were mixed with SDS loading buffer for 7 min at 23 °C. Electrophoresis was performed with a 1.5% (*w*/*v*) Tris-glycine, 0.1% (*v*/*v*) SDS, agarose gel (SeaKem Gold PFGE agarose, *Lonza*, Rockland, ME, USA), at 120 V for 40 min. Proteins were transferred onto nitrocellulose membrane by western transfer at 1 A for 1 h at 4 °C in tris-glycine/methanol buffer. Membranes were then blocked with a 5% milk solution and immunoblotted with Sup35-antibody. The membrane was then imaged with the BioRad Imager standard protocol (*BioRad*, Hercules, CA, USA).

Sodium dodecyl sulfate-polyacrylaminde gel electrophoresis (SDS-PAGE) was used to confirm repression of *SIS1*. Cell pellets of 1 OD_600_ were collected and resuspended in 0.2 M NaOH and vortexed briefly, then allowed to incubate at 23 °C for 5 min. Cells were centrifuged at 14,500 rpm on a tabletop centrifuge for 1 min and supernatant removed. Pellets were resuspended in SDS-PAGE sample buffer and boiled for 5 min. Electrophoresis was performed with a 12.5% polyacrylamide gel at 120 V. Proteins were immunoblotted using same method as SDD-AGE procedure, using Sis1 antibody instead of Sup35.

### 2.3. SIS1 Repression and Plasmid-Shuffling

25 mL liquid cultures of polyQX strains of the 74D-694 background expressing Sis1 on the plasmid p3617 (*TETr-Sis1)* were maintained in log phase growth in synthetic medium lacking uracil to maintain the presence of the polyQX plasmids. Cultures were reinoculated and pellets of 1 OD_600_ and 4–8 OD_600_ collected every 10 generations, for a total of 20–100 generations. *TETr-Sis1* was repressed by addition of the tetracycline analog doxycycline at 5 µg/mL with each reinoculation.

PolyQX strains in the 74D-694 background expressing Sis1 on the plasmid p1849 ([*SIS1*-Sis1, *TRP1*]) were transformed by either plasmid bearing full-length Sis1 ([*SIS1*-Sis1, *HIS3*]) or Sis1-∆G/F ([*SIS1*-Sis1, *HIS3*]) and at least 10 transformants were patched onto 5-FAA media to counterselect against the *TRP1* marker of p1849. Patches were repeatedly patched onto 5-FAA and then tested for Trp auxotrophy.

## 3. Results

### 3.1. Confirming PolyQX Aggregation and Hsp104-Dependent Propagation

To begin to examine the potential for a JDP requirement for synthetic prion propagation, we first needed to establish whether polyQX constructs would form stably propagating aggregates in our yeast strains. Constructs are denoted as in Alexandrov et al. by the overall length of the QQQXQ repeat region which sometimes ends with an additional terminal Q, followed by the letter Q and single letter code for the heterologous residue [41]. For example, 101QM represents a QQQMQ repeat domain of 101 total residues, N-terminally fused to the M and C domains of Sup35. The 85QQ construct is distinct in that there is no heterologous residue but rather a stretch of 85 Q residues.

Only a subset of the plasmid-based constructs described in Alexandrov et al., 2012 were gifted and successfully transformed both *E. coli* and our yeast strains. Of those, some—those bearing L, E, and P—do not form amyloid as previously published [41]. However, following transformation with plasmids expressing each chimera, we were able to isolate strains with stably propagating aggregates for 76QY, 101QM, 85QQ, 81QF, 81QS, and 91QV (Figure 1B). Additionally, as previously shown by Alexandrov et al. for the 76QY construct [41], SDS-resistant polymers of 101QM, 85QQ, 81QF, 81QS, and 91QV are lost upon treatment with GdnHCl (Figure 1C, only 91QV shown), indicating that they depend upon Hsp104 and propagate in a prion-like manner.

### 3.2. Assaying for Sis1-Depdendence

We next wanted to assess whether these polyQX synthetic prions require Sis1 activity for propagation. Although Sis1 is an essential protein in yeast, its expression can be significantly reduced and only higher levels of Sis1 are necessary for prion maintenance [49]. This approach has been exploited to determine the relative requirements for Sis1 among prions and prion variants by analyzing the kinetics of prion loss [24,25,50]. We chose the 74D-694 yeast genetic background specifically for these experiments due to our previous observations of an unidentified genetic polymorphism that affects the curing of some prions by *SIS1* repression in our W303 strains [50]. As an additional consideration, the prion [*PIN*^+^] (also called [*RNQ*^+^]) is known to induce the formation of other prions and affects polyQ aggregate formation and toxicity [5,52,53,54,55,56,57]. To eliminate any complication in interpretation of the results that could be caused by cross-seeding or another interaction with a second yeast prion, we utilized exclusively [*psi*^−^] and [*pin*^−^] strains for these experiments.

We again employed a system placing *SIS1* under the control of the *tet^R^* promoter (*TETr*), which allows the repression of Sis1 synthesis upon addition of doxycycline [49]. Cells were grown in continuous log phase in liquid medium with agitation for 20 generations with or without doxycycline. Culture experiments with doxycycline were conducted in triplicate for each polyQX construct. Aliquots of each culture were taken periodically for analysis by SDS-PAGE and SDD-AGE. This timeframe (20 generations) was chosen because it allows for the curing of most yeast prions under these same conditions including [*URE3*], [*SWI*^+^], [*PIN*^+^]/[*RNQ*^+^], and weak variants of [*PSI*^+^] [24,25,49,50]. Only strong [*PSI*^+^] variants, which are exceptionally insensitive to Sis1 activity relative to other studied prions, persist past this point [24,50].

**Figure 2:**
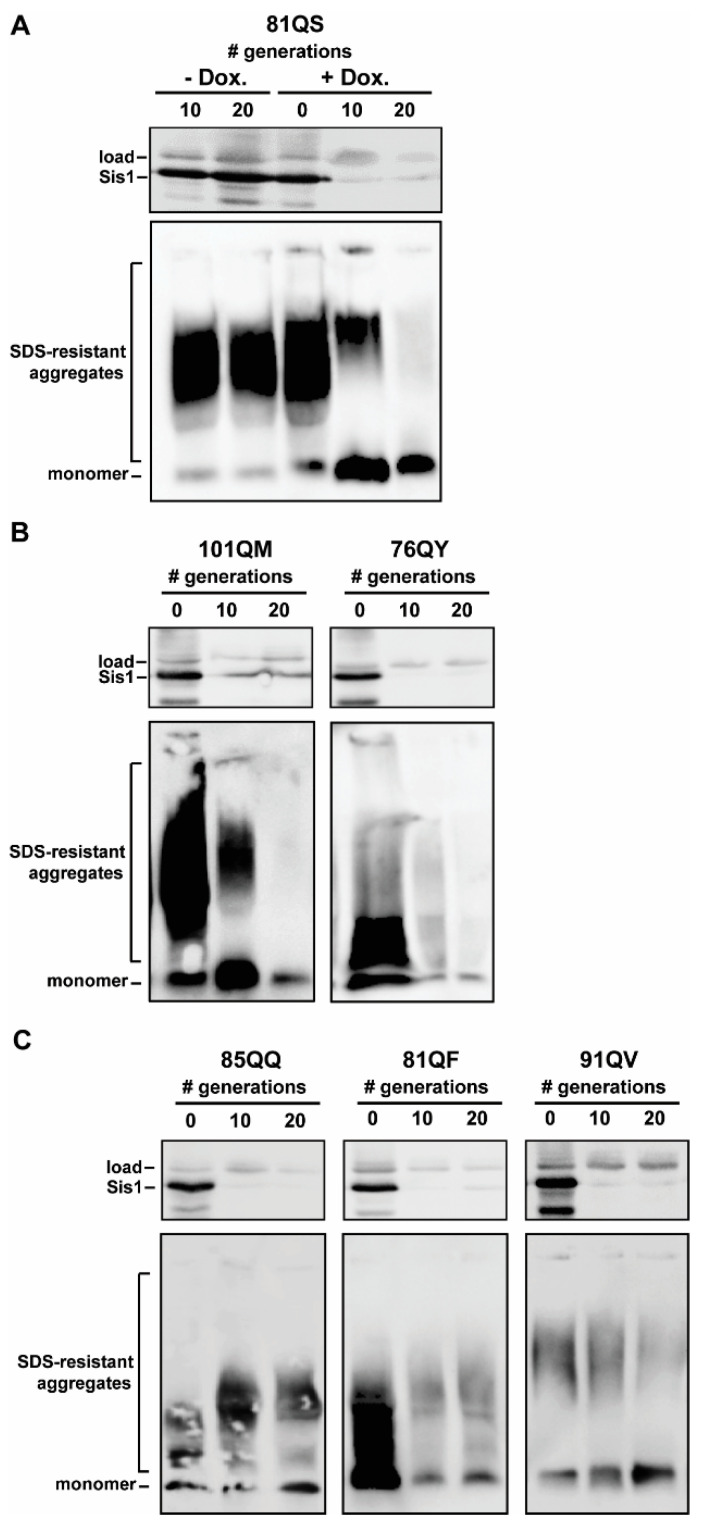
PolyQX synthetic prions are sensitive to Sis1 expression. Time courses of *SIS1*-repression of cells bearing distinct polyQX prions were conducted in triplicate with similar results; representative results are shown. (**A**–**C**) Cells were harvested after the indicated number of generations of growth in the absence (−Dox.) or presence (+Dox.) of doxycycline and pellets collected for analysis. Lysates were resolved by SDS-PAGE (**top panels**) and SDD-AGE (**bottom panels**). Sup35 and Sis1 were visualized by immunoblotting with Sup35- and Sis1-specific antibodies. A band cross-reacting with the Sis1 antibody is shown as a loading control. (**A**,**B**) PolyQX prion aggregates of 81QS, 101QM, and 76QY are lost following 20 generations of growth on doxycycline. (**C**) Detergent-resistant aggregates persist to 20 generations for 85QQ, 81QF, and 91QV.

All six tested polyQX prions were invariably maintained in the absence of doxycycline as shown for 81QS (Figure 2A), indicative of their stable propagation in haploid yeast populations in liquid culture. *SIS1* repression, on the other hand, severely affected polyQX propagation of 81QS (Figure 2A), as well as 101QM and 76QY (Figure 2B); no detergent-resistant aggregates were observed after 20 generations, indicating the complete loss of the prions from the population. The kinetics of prion loss were similar to those observed for [*URE3*], [*RNQ*^+^], and [*SWI*^+^], indicating a relatively high sensitivity to the loss of Sis1 function [24,25]. Notably, these observations indicate that Sis1 is involved in the propagation of amyloid aggregates that are distinct from native prions and that have notably low-complexity PrDs with only two residue identities present within each.

**Figure 3:**
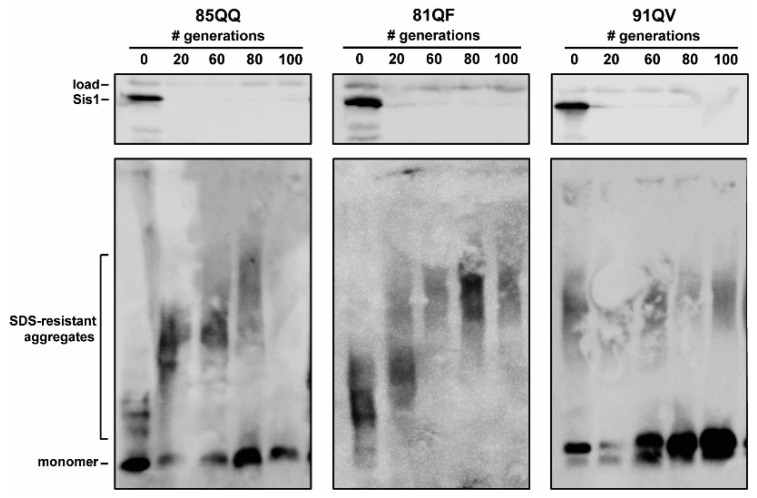
Time courses of *SIS1*-repression of cells bearing distinct polyQX prions of 85QQ, 81QF, and 91QV for 100 generation were conducted in triplicate with similar results; representative results are shown. Cells were harvested after the indicated number of generations of growth in the presence of doxycycline and pellets collected for analysis. Lysates were resolved by SDS-PAGE (**top panels**) and SDD-AGE (**bottom panels**). Sup35 and Sis1 were visualized by immunoblotting with Sup35- and Sis1-specific antibodies. A band cross-reacting with the Sis1 antibody is shown as a loading control.

In contrast, three constructs—85QQ, 81QF, and 91QV—had detectable aggregates that persisted into the 20th generation (Figure 2C). Notably 81QF and 91QV appeared to have fewer aggregates by generation 20 while an increase in aggregate size was apparent for 85QQ, indicating that *SIS1* repression may have caused a fragmentation defect which may result in curing if a longer timescale was used. To test this, we repeated the *SIS1* repression experiments with each of these three constructs, again in triplicate, but this time culturing the cells until 100 generations and collecting cells for analysis every 20 generations. Importantly, this timeframe (100 generations) is past the point of curing for any known yeast prion as other studies have found that the strong [*PSI*^+^] variants persist out to approximately 50–80 generations under the same conditions [24,50]. On this considerably longer timescale, 85QQ was cured, however 81QF and 91QV had detectable aggregates out to 100 generations (Figure 3). Notably a shift to larger aggregate size was again noticeable for 85QQ prior to complete curing, as well as for 81QF, despite the lack of complete curing. An increase in monomeric protein was apparent for 91QV, but otherwise no change in detergent-resistant aggregates was apparent, despite the clear repression of Sis1 for this extremely long timescale of growth.

### 3.3. Pilot Work on Sis1-Domain Dependence and Secondary JDP Requirements

Different native prions and prion variants exhibit diverse requirements for specific Sis1 domains and sequence regions [22,30]. Most notable in terms of its importance, is the glycine-phenylalanine-rich (G/F) region which sits adjacent to the J domain of Sis1. This region is critically important for the propagation of all known variants of [*PIN*^+^]/[*RNQ*^+^] without exception [22,26,29]. To begin to determine if specific regions of Sis1 may play a part in polyQX prion propagation, we conducted a pilot experiment utilizing a commonly used Sis1 protein construct lacking the G/F region (Sis1-∆G/F) and testing the ability of this construct to propagate polyQX prions by yeast plasmid shuffling.

**Figure 4:**
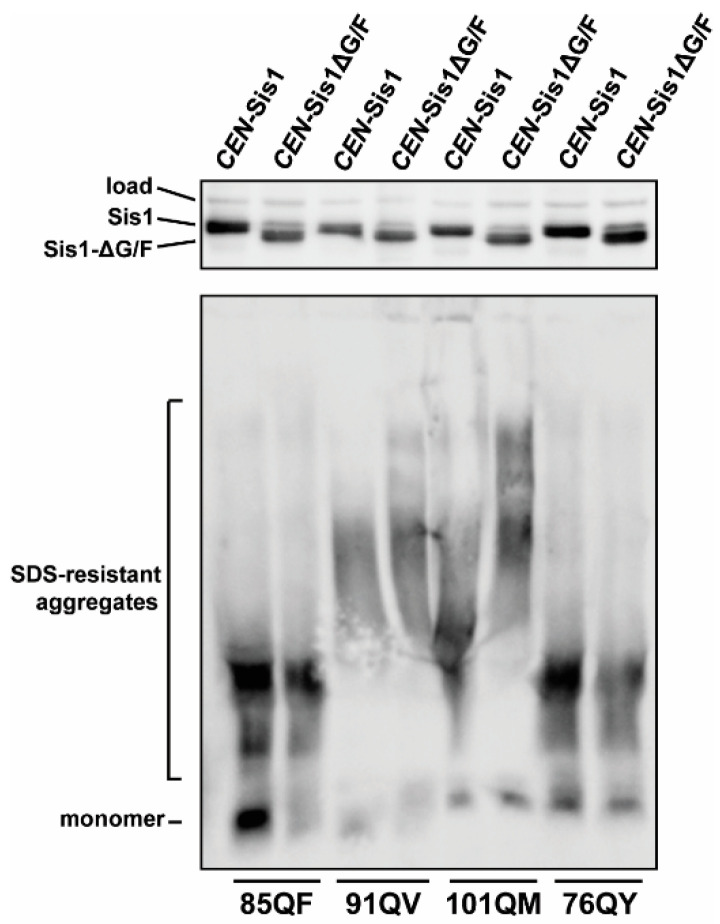
Plasmid shuffling experiments to test for a G/F region requirement of Sis1 by polyQX synthetic prions. PolyQX prion-bearing cells were transformed by plasmids expressing Sis1 or an internal deletion (Sis1ΔG/F) and subjected to plasmid shuffling. Cell lysates were prepared from *sis1*-Δ cells expressing either wild-type Sis1 or Sis1ΔG/F from a plasmid and were subjected to SDS-PAGE (**top panel**) and SDD-AGE (**bottom panel**) followed by immunoblot analysis with Sis1 and Sup35 antibodies. A band cross-reacting with the Sis1 antibody is shown as a loading control. In this case, only one shuffling attempt was successful for each construct.

We utilized a [*psi*^−^], [*pin*^−^] *sis1*-Δ 74D-694 strain expressing Sis1 from a *URA3*-marked plasmid. This allows a plasmid expressing Sis1-∆G/F to be shuffled in to replace the wild-type expressing plasmid without prion or viability loss due to a temporary lack of Sis1 expression. Following transformation by the Sis1-∆G/F plasmid, and subsequent growth on 5-fluoroorotic acid (5-FOA) which counter-selects against the *URA3* gene [58], cells were passaged on solid medium twice and then the presence of the prion is again assayed. A full-length Sis1 expressing plasmid marked with *TRP1* was used as a control to account for random prion loss during experimental manipulations. Successful shuffling was ascertained by SDS-PAGE followed by immunoblotting with Sis1 antibody that recognizes the smaller Sis1-∆G/F protein construct. For reasons which are unclear to us, shuffling experiments were repeatedly unsuccessful for 81QS and 81QF; it is possible that these constructs could become toxic in the absence of the G/F region, but this is only speculation. We were able to successfully shuffle in the Sis1-∆G/F construct in strains harboring 85QQ, 91QV, 101QM, and 76QY at least once. In each case, the prion was maintained (Figure 4), indicating that these constructs do not share the strict requirement for the G/F region that [*PIN*^+^]/[*RNQ*^+^] exhibits, however these results should be regarded as preliminary given they are single observations. A shift to higher molecular weights, again indicative of potential fragmentation defect, was apparent for 101QM despite some distortion in the blot. This shift was confirmed upon repetition of the gel/blot.

Finally, we conducted one additional pilot investigation to explore the possibility that polyQX synthetic prions might exhibit secondary JDP requirements in addition to Sis1. We turned our attention to the JDP Swa2 which is essential for the propagation of the prion [*URE3*], specifically. Swa2 is the yeast homolog of the mammalian protein auxilin, which is involved in the disassembling of the clathrin lattice following endocytosis [59]. Swa2 has N-terminal clathrin-binding domains, along with C-terminal J and TPR domains [60]; we previously determined that the C-terminal domains are both essential for [*URE3*] propagation while the N-terminal clathrin-binding domains not necessary, implying that clathrin binding is not a vital component of Swa2′s secondary requirement by the prion [27]. We proposed a current model for Swa2 function in [*URE3*] prion propagation in which the TPR region is involved in binding a multi-protein complex which includes Hsp90 and a cochaperone and is responsible for delivering additional [*URE3*] to Hsp70 which can then recruit Hsp104 for disaggregation [61]. Secondary JDP requirements have only been explored for a few prions and have likely never been investigated for synthetic prions or other polyQ constructs.

**Figure 5:**
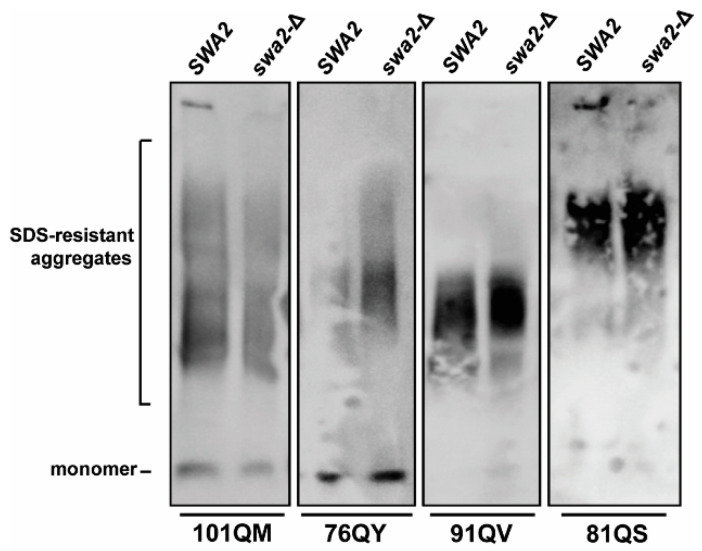
The JDP Swa2 is dispensable for the propagation of at least four polyQX prions. Haploid [*psi*^−^], [*pin*^−^] strains bearing four distinct polyQX prions were crossed to a [*psi*^−^], [*pin*^−^] strain bearing a deletion of the *SWA2* gene (*swa2-∆*). Following sporulation and tetrad dissection, *swa2-*∆ F1 haploids as well as F1 haploids which maintained the *SWA2* gene were assayed for the maintenance of each polyQX prion by SDD-AGE followed by immunoblotting with Sup35 antibody. Representative examples are shown from *n* ≥ 5 in each case.

To examine whether polyQX synthetic prions might also exhibit a secondary JDP requirement for Swa2 in addition to the requirement for Sis1, a W303-derived *swa2*-∆, [*psi*^−^], [*pin*^−^] strain was crossed with W303 [*psi*^−^], [*pin*^−^] strains bearing polyQX aggregates. We used the W303 genetic background in this case due to high sporulation rate which enables many crosses to be completed quickly as well as the availably of the well-characterized *swa2*-∆ strain [23,27]. Following diploid sporulation and subsequent tetrad dissection, the presence or absence of the polyQX aggregates in the resulting haploids was assayed by SDD-AGE in both *SWA2* and *swa2*-∆ progeny (*n* ≥ 5). We were able to isolate polyQX-maintaining haploids that stably propagated the prions in strains bearing 101QM, 76QY, 91QV, and 81QS (Figure 5) but were unable to confirm stable propagation of 85QQ and 81QF due to apparent prion instability during mating, sporulation, and/or germination. Despite this, these data indicate that Swa2 is not required for at least four of these synthetic prions, congruent with its dispensability for [*PSI*^+^] and [*PIN*^+^]/[*RNQ*^+^] propagation.

## 4. Discussion

The goal of the work described here was to gain a better understanding of the relationship between chaperone function and prion propagation with respect to the composition of a PrD. We are interested in the following question: Do JDPs recognize specific sequence elements within naturally occurring yeast prion PrDs, or are they acting more broadly, perhaps recognizing a common amyloid or amyloid-forming structure? While the results presented here are limited in scope and based solely on electrophoresis assays, they constitute a significant step toward understanding how amino acid sequences within prion domains affect amyloid and chaperone behavior in vivo.

### 4.1. Impact of SIS1 Repression

Our work constituted a logical next step from the initial investigations of Alexandrov et al. [41]. Those investigators demonstrated that one polyQX prion depended upon Hsp104 and justifiably speculated that Sis1/Hsp70 are likely involved. Here, we revealed a requirement of Sis1 for the propagation of four of these prions with synthetic, and extremely low-complexity, PrDs. These results demonstrate that these synthetic prions require Sis1 activity for propagation in cell populations like other native prions, implying that Sis1 does not likely recognize specific sequence elements of PrDs. Prion recognition by chaperones is therefore likely primarily due to amino acid composition of the PrD and, as suggested by Alexandrov et al., individual residues may be enough to act as recognition elements [41]. Our data further support these assertions.

Surprisingly, we uncovered dramatically different responses of synthetic polyQX prions to Sis1 repression, with some prion constructs, 81QF and 91QV, persisting in Sis1-depleted cell populations longer than any known native prion. Hsp104-dependent, yet Sis1-independent, prion propagation is unprecedented as Sis1 has been previously found to be required for all prions for which there is data [24,25,26,29,50]. The apparent Sis1-independence of the 91QV aggregates will need to be investigated further and it is additionally unclear if a longer culture time would eventually cure 81QF, however practical issues make these long cell culture experiments extremely difficult. Maintaining Sis1 repression, without the appearance of suppressor mutations that restore Sis1 expression for 100 generations, is significantly challenging and required many attempts. Because Sis1 is essential for cell viability, irrefutably demonstrating Sis1-independence is not straightforward. Additional tests using poorly functional Sis1 constructs, or extragenic complementation possibly with a construct like Ydj1-134G^70^ → N that supports cell viability at a reduced level [62], may help to further establish whether propagation is truly Sis1-independent. Regardless, these results revealed that chaperone requirements may differ dramatically among amyloid aggregates, perhaps in unanticipated ways.

### 4.2. Sis1 Domain Requirements

Native prions vary with regard to the domains of Sis1 necessary for propagation and the construct Sis1-∆G/F has been the most studied in this regard [22]. All known variants of the yeast prion [*PIN*^+^]/[*RNQ*^+^] absolutely require the G/F region of Sis1 [12,22,29,63]. In contrast, the prions formed by the constructs 85QQ, 91QV, 101QM, and 76QY—constituting all four for which we were able to gather data—were all maintained by Sis1-∆G/F, similar to weak and strong variants of [*PSI^+^*]. As noted above, 91QV may actually be Sis1-independent; these observations are also consistent with that hypothesis. 101QM, however, demonstrated a shift toward higher molecular weight aggregates, indicating a difference in the activity of Sis1 and Sis1-∆G/F in maintaining propagation of these aggregates. 101QM was also lost after 20 generations of *SIS1* repression. Combined, our observations indicate that 101QM, and possibly 81QS, are the most Sis1 sensitive constructs in our data set. These results further highlight the notion that synthetic and native prions exhibit variations in chaperone requirements. Additional work using polyQX prions with normalized repeat lengths (discussed further below) would more clearly illuminate the role of individual residues as the determinants of this diversity.

### 4.3. Secondary JDP Requirements

There are 13 JDPs at least partially present in the yeast cytosol and to date, four—Sis1, Ydj1, Swa2, and Apj1—have been implicated in prion biology [26,27,64,65]. To the best of our knowledge, secondary JDP requirements for artificial polyQ or polyQX aggregates, or any other synthetic prions for that matter, have not been explored prior to this investigation. For native prions, secondary JDP requirements have been determined for two prions: in addition to Sis1, [*URE3*] requires the JDP Swa2 while [*SWI*^+^] requires the JDP Ydj1 [25,27]. In contrast, weak and strong variants of [*PSI^+^*], as well as at least one strong variant of [*PIN*^+^]/[*RNQ*^+^], require only Sis1 [24,66]. It remains to be determined whether these requirements are due to specific protein–protein interactions, as we have suggested for [*URE3*] and Swa2, or rather are due to some common physical characteristic of the amyloid. Here, we eliminated a secondary requirement for Swa2 for four synthetic prions, including 101QM and 81QS, with no variation among the four tested and no phenotypes apparent as a result of *SWA2* deletion. These results are consistent with the hypothesis that Swa2 acts in [*URE3*] propagation, specifically, due to interactions with Hsp90 rather than acting as some kind of general amyloid recognition factor [61]. Because Apj1 is not required for the propagation of any known prions, but rather has only so far been implicated in prion curing, we would predict that polyQX prions would propagate in an *apj1*-Δ strain, whereas *ydj1*-Δ is more likely to exhibit prion propagation defects. Further work will be necessary to rule in or rule out other JDP interactions with these and other synthetic prions.

### 4.4. Amino Acid Composition, Current Limitations, and Future Directions

Alexandrov et al. created this set of polyQX prions with the goal of testing differences in the functionality of specific residue identities in PrDs and concluded that some residues promoted aggregated fragmentation in vivo [41]. There is considerable overlap between those amino acids that Alexandrov et al. suggested promote efficient fragmentation in polyQ constructs and those that Maclea et al. found to be both overrepresented in yeast PrDs and most promoting of prion maintenance. Most notably, F, W, and Y were the most prion-promoting amino acids in both scenarios, and N, Q, S, and T were mildly prion-promoting, although this set exhibited greater differences in aggregate size [41,42]. Finally, several amino acids did not exhibit any polyQ aggregation when placed in the polyQX context, and were among those that had a negative prion propensity score, specifically R, L, P, and E. These results together indicate that many amino acids have similar effects on both yeast prion and polyQ aggregate maintenance, further supporting the use of this model system to study amyloid behavior in vivo.

However, a major limitation of this system is that the current set of polyQX protein constructs have variations in the lengths of the QX repeat, introducing a confounding variable that significantly complicates direct comparisons based on residue identities. While this initial set has been useful for allowing us to gather proof-of-principle data, and to make some discoveries, it will be insufficient to address the greater question of the role of individual amino acid identities in driving chaperone interactions and in vivo amyloid behavior—that would require normalization of repeat lengths. For this reason, we refrain from speculating on this basis regarding amino acid identities for our own results. Findings for each individual polyQX construct must be viewed independently as each represents a unique synthetic prion. Thus, these experiments are not sufficient to compare requirements for amino acids within the PrD region of proteins but rather serve as a pilot exploration of JDP requirements for independent synthetic polyQX prions.

## 5. Conclusions

The future creation of a new set of length-normalized constructs should help to support or refute hypotheses about particular amino acid identities and their correlations with chaperone requirements and offer many opportunities to dissect the complexities of prion biology. Our findings that Sis1 is indeed necessary for some prions formed from extremely low-complexity domains supports the idea that because JDPs often act as targeting factors for Hsp70s, they may constitute the first response of the chaperone systems to the presence of amyloid. Thus, this current work provides new insight into the physical basis of chaperone-amyloid interactions and contributes to our growing understanding of how amino acid sequences form stable amyloids in vivo in a chaperone-dependent manner.

## Data Availability

Not applicable.

## References

[1] Prusiner S.B. (2013). Biology and Genetics of Prions Causing Neurodegeneration. Annu. Rev. Genet..

[2] Wickner R.B. (1994). [URE_3_] as an Altered URE_2_ Protein: Evidence for a Prion Analog in *Saccharomyces cerevisiae*. Science.

[3] Wickner R.B., Masison D.C., Edskes H.K. (1995). [PSI] and [URE_3_] as yeast prions. Yeast.

[4] Derkatch I.L., O Chernoff Y., Kushnirov V.V., Inge-Vechtomov S.G., Liebman S.W. (1996). Genesis and variability of [PSI] prion factors in Saccharomyces cerevisiae. Genetics.

[5] Derkatch I.L., E Bradley M., Hong J.Y., Liebman S.W. (2001). Prions Affect the Appearance of Other Prions: The Story of [PIN+]. Cell.

[6] Alberti S., Halfmann R., King O., Kapila A., Lindquist S. (2009). A Systematic Survey Identifies Prions and Illuminates Sequence Features of Prionogenic Proteins. Cell.

[7] Du Z., Park K.-W., Yu H., Fan Q., Li L. (2008). Newly identified prion linked to the chromatin-remodeling factor Swi1 in Saccharomyces cerevisiae. Nat. Genet..

[8] Liebman S.W., Chernoff Y.O. (2012). Prions in yeast. Genetics.

[9] Verma A.K., Diwan D., Raut S., Dobriyal N., Brown R.E., Gowda V., Hines J.K., Sahi C. (2017). Evolutionary Conservation and Emerging Functional Diversity of the Cytosolic Hsp70:J Protein Chaperone Network of *Arabidopsis thaliana*. G3 Genes Genomes Genet..

[10] Verma A.K., Tamadaddi C., Tak Y., Lal S.S., Cole S.J., Hines J.K., Sahi C. (2019). The expanding world of plant J-domain proteins. Crit. Rev. Plant Sci..

[11] Stein K.C., Bengoechea R., Harms M.B., Weihl C.C., True H.L. (2014). Myopathy-causing Mutations in an HSP40 Chaperone Disrupt Processing of Specific Client Conformers. J. Biol. Chem..

[12] Lopez N., Aron R., Craig E.A. (2003). Specificity of class II Hsp40 Sis1 in maintenance of yeast prion [RNQ+]. Mol. Biol. Cell.

[13] Reidy M., Masison D.C. (2014). Yeast prions help identify and define chaperone interaction networks. Curr. Pharm. Biotechnol..

[14] Reidy M., Sharma R., Roberts B.-L., Masison D.C. (2016). Human J-protein DnaJB6b Cures a Subset of Saccharomyces cerevisiae Prions and Selectively Blocks Assembly of Structurally Related Amyloids. J. Biol. Chem..

[15] Sporn Z.A., Hines J.K. (2015). Hsp40 function in yeast prion propagation: Amyloid diversity necessitates chaperone functional complexity. Prion.

[16] Kampinga H.H., Andreasson C., Barducci A., Cheetham M.E., Cyr D., Emanuelsson C., Genevaux P., Gestwicki J.E., Goloubinoff P., Huerta-Cepas J. (2018). Function, evolution, and structure of J-domain proteins. Cell Stress Chaperones.

[17] Tuite M.F., Mundy C.R., Cox B.S. (1981). Agents that cause a high frequency of genetic change from [psi+] to [psi-] in Saccharomyces cerevisiae. Genetics.

[18] Grimminger V., Richter K., Imhof A., Buchner J., Walter S. (2004). The Prion Curing Agent Guanidinium Chloride Specifically Inhibits ATP Hydrolysis by Hsp104. J. Biol. Chem..

[19] Wickner R.B., Edskes H.K., Shewmaker F., Nakayashiki T. (2007). Prions of fungi: Inherited structures and biological roles. Nat. Rev. Genet..

[20] Winkler J., Tyedmers J., Bukau B., Mogk A. (2012). Hsp70 targets Hsp100 chaperones to substrates for protein disaggregation and prion fragmentation. J. Cell Biol..

[21] Satpute-Krishnan P., Langseth S.X., Serio T.R. (2007). Hsp104-Dependent Remodeling of Prion Complexes Mediates Protein-Only Inheritance. PLoS Biol..

[22] Killian A.N., Hines J.K. (2018). Chaperone functional specificity promotes yeast prion diversity. PLoS Pathog..

[23] Sahi C., Craig E.A. (2007). Network of general and specialty J protein chaperones of the yeast cytosol. Proc. Natl. Acad. Sci. USA.

[24] Higurashi T., Hines J.K., Sahi C., Aron R., Craig E.A. (2008). Specificity of the J-protein Sis1 in the propagation of 3 yeast prions. Proc. Natl. Acad. Sci. USA.

[25] Hines J.K., Li X., Du Z., Higurashi T., Li L., Craig E.A. (2011). [SWI], the prion formed by the chromatin remodeling factor Swi1, is highly sensitive to alterations in Hsp70 chaperone system activity. PLoS Genet..

[26] Sondheimer N., Lopez N., Craig E.A., Lindquist S. (2001). The role of Sis1 in the maintenance of the [RNQ+] prion. EMBO J..

[27] Troisi E.M., Rockman M.E., Nguyen P.P., Oliver E.E., Hines J.K. (2015). Swa2, the yeast homolog of mammalian auxilin, is specifically required for the propagation of the prion variant [URE3-1]. Mol. Microbiol..

[28] Harris J.M., Nguyen P.P., Patel M.J., Sporn Z.A., Hines J.K. (2014). Functional diversification of hsp40: Distinct j-protein functional requirements for two prions allow for chaper-one-dependent prion selection. PLoS Genet..

[29] Stein K.C., True H.L. (2014). Extensive diversity of prion strains is defined by differential chaperone interactions and distinct amyloi-dogenic regions. PLoS Genet..

[30] Killian A.N., Miller S.C., Hines J.K. (2019). Impact of Amyloid Polymorphism on Prion-Chaperone Interactions in Yeast. Viruses.

[31] Taylor K.L., Cheng N., Williams R.W., Steven A.C., Wickner R.B. (1999). Prion Domain Initiation of Amyloid Formation in Vitro from Native Ure2p. Science.

[32] Glover J.R., Kowal A.S., Schirmer E.C., Patino M.M., Liu J.-J., Lindquist S. (1997). Self-Seeded Fibers Formed by Sup35, the Protein Determinant of [PSI+], a Heritable Prion-like Factor of S. cerevisiae. Cell.

[33] Vitrenko Y.A., Pavon M.E., Stone S.I., Liebman S.W. (2007). Propagation of the [PIN+] prion by fragments of Rnq1 fused to GFP. Curr. Genet..

[34] Du Z., Crow E.T., Kang H.S., Li L. (2010). Distinct Subregions of Swi1 Manifest Striking Differences in Prion Transmission and SWI/SNF Function. Mol. Cell. Biol..

[35] Ross E.D., Baxa U., Wickner R.B. (2004). Scrambled Prion Domains Form Prions and Amyloid. Mol. Cell. Biol..

[36] Ross E.D., Edskes H.K., Terry M.J., Wickner R.B. (2005). Primary sequence independence for prion formation. Proc. Natl. Acad. Sci. USA.

[37] Ross E.D., Toombs J.A. (2010). The effects of amino acid composition on yeast prion formation and prion domain interactions. Prion.

[38] Cascarina S.M., Paul K.R., Machihara S., Ross E.D. (2018). Sequence features governing aggregation or degradation of prion-like proteins. PLoS Genet..

[39] Toombs J., McCarty B.R., Ross E.D. (2010). Compositional Determinants of Prion Formation in Yeast. Mol. Cell. Biol..

[40] Alexandrov I.M., Vishnevskaya A.B., Ter-Avanesyan M.D., Kushnirov V.V. (2008). Appearance and Propagation of Polyglutamine-based Amyloids in Yeast: TYROSINE RESIDUES ENABLE POLYMER FRAGMENTATION. J. Biol. Chem..

[41] Alexandrov A.I., Polyanskaya A.B., Serpionov G.V., Ter-Avanesyan M.D., Kushnirov V.V. (2012). The Effects of Amino Acid Composition of Glutamine-Rich Domains on Amyloid Formation and Fragmentation. PLoS ONE.

[42] MacLea K.S., Paul K.R., Ben-Musa Z., Waechter A., Shattuck J.E., Gruca M., Ross E.D. (2015). Distinct Amino Acid Compositional Requirements for Formation and Maintenance of the [*PSI*^+^] Prion in Yeast. Mol. Cell. Biol..

[43] Hines J.K., Craig E.A. (2011). The sensitive [SWI(+)] prion: New perspectives on yeast prion diversity. Prion.

[44] Takahashi T., Katada S., Onodera O. (2010). Polyglutamine Diseases: Where does Toxicity Come from? What is Toxicity? Where are We Going?. J. Mol. Cell Biol..

[45] Krobitsch S., Lindquist S. (2000). Aggregation of huntingtin in yeast varies with the length of the polyglutamine expansion and the expression of chaperone proteins. Proc. Natl. Acad. Sci. USA.

[46] DePace A.H., Santoso A., Hillner P., Weissman J.S. (1998). A Critical Role for Amino-Terminal Glutamine/Asparagine Repeats in the Formation and Propagation of a Yeast Prion. Cell.

[47] Osherovich L.Z., Cox B.S., Tuite M.F., Weissman J.S. (2004). Dissection and Design of Yeast Prions. PLoS Biol..

[48] Yan W., Craig E.A. (1999). The Glycine-Phenylalanine-Rich Region Determines the Specificity of the Yeast Hsp40 Sis1. Mol. Cell. Biol..

[49] Aron R., Higurashi T., Sahi C., Craig E.A. (2007). J-protein co-chaperone Sis1 required for generation of [RNQ+] seeds necessary for prion propagation. EMBO J..

[50] Hines J.K., Higurashi T., Srinivasan M., Craig E.A. (2011). Influence of prion variant and yeast strain variation on prion-molecular chaperone requirements. Prion.

[51] Mumberg D., Müller R., Funk M. (1995). Yeast vectors for the controlled expression of heterologous proteins in different genetic backgrounds. Gene.

[52] Derkatch I.L., Bradley M.E., Masse S.V., Zadorsky S.P., Polozkov G.V., Inge-Vechtomov S.G., Liebman S.W. (2000). Dependence and independence of [PSI(+)] and [PIN(+)]: A two-prion system in yeast?. EMBO J..

[53] Derkatch I.L., Uptain S.M., Outeiro T.F., Krishnan R., Lindquist S.L., Liebman S.W. (2004). Effects of Q/N-rich, polyQ, and non-polyQ amyloids on the de novo formation of the [PSI+] prion in yeast and aggregation of Sup35 in vitro. Proc. Natl. Acad. Sci. USA.

[54] Yang Z., Hong J.Y., Derkatch I.L., Liebman S.W. (2013). Heterologous Gln/Asn-Rich Proteins Impede the Propagation of Yeast Prions by Altering Chaperone Availability. PLoS Genet..

[55] Kurahashi H., Ishiwata M., Shibata S., Nakamura Y. (2008). A Regulatory Role of the Rnq1 Nonprion Domain for Prion Propagation and Polyglutamine Aggregates. Mol. Cell. Biol..

[56] Meriin A.B., Zhang X., He X., Newnam G.P., Chernoff Y.O., Sherman M.Y. (2002). Huntington toxicity in yeast model depends on polyglutamine aggregation mediated by a prion-like protein Rnq1. J. Cell Biol..

[57] Osherovich L.Z., Weissman J.S. (2001). Multiple Gln/Asn-Rich Prion Domains Confer Susceptibility to Induction of the Yeast [PSI] Prion. Cell.

[58] Sikorski R.S., Boeke J.D. (1991). In vitro mutagenesis and plasmid shuffling: From cloned gene to mutant yeast. Methods Enzymol..

[59] Gall W.E., Higginbotham M.A., Chen C.-Y., Ingram M.F., Cyr D.M., Graham T.R. (2000). The auxilin-like phosphoprotein Swa2p is required for clathrin function in yeast. Curr. Biol..

[60] Xiao J., Kim L.S., Graham T.R. (2006). Dissection of Swa2p/Auxilin Domain Requirements for Cochaperoning Hsp70 Clathrin-uncoating Activity In Vivo. Mol. Biol. Cell.

[61] Oliver E.E., Troisi E.M., Hines J.K. (2017). Prion-specific Hsp40 function: The role of the auxilin homolog Swa2. Prion.

[62] Schilke B.A., Ciesielski S., Ziegelhoffer T., Kamiya E., Tonelli M., Lee W., Cornilescu G., Hines J.K., Markley J.L., Craig E.A. (2017). Broadening the functionality of a J-protein/Hsp70 molecular chaperone system. PLoS Genet..

[63] Stein K.C., True H.L. (2014). Structural variants of yeast prions show conformer-specific requirements for chaperone activity. Mol. Microbiol..

[64] Moriyama H., Edskes H.K., Wickner R.B. (2000). [URE3] Prion Propagation in *Saccharomyces cerevisiae*: Requirement for Chaperone Hsp104 and Curing by Overexpressed Chaperone Ydj1p. Mol. Cell. Biol..

[65] Berger S.E., Nolte A.M., Kamiya E., Hines J.K. (2019). Three J-proteins impact Hsp104-mediated variant-specific prion elimination: A new critical role for a low-complexity domain. Curr. Genet..

[66] Astor M.T., Kamiya E., Sporn Z.A., Berger S., Hines J.K. (2018). Variant-specific and reciprocal Hsp40 functions in Hsp104-mediated prion elimination. Mol. Microbiol..

